# Classified metabolic power-based measures in professional football players: comparison between playing positions and match period

**DOI:** 10.1186/s13102-022-00541-y

**Published:** 2022-07-30

**Authors:** Zeki Akyildiz, Erhan Çene, Coşkun Parim, Onat Çetin, Çağatay Turan, Yılmaz Yüksel, Rui Silva, Ana Filipa Silva, Hadi Nobari

**Affiliations:** 1 Sports Science Department, Gazi University, Ankara, Turkey; 2 Department of Statistics, Yildiz Technical University, Istanbul, Turkey; 3 Faculty of Sports Sciences, Department of Coaching Education, Yalova University, Yalova, Turkey; 4 Konya Spor Football Club, Konya, Turkey; 5 Sports Science Department, Anadolu University, 26170 Eskisehir, Turkey; 6 Escola Superior Desporto e Lazer, Instituto Politécnico de Viana Do Castelo, Rua Escola Industrial e Comercial de Nun’Álvares, 4900-347 Viana do Castelo, Portugal; 7 Research Center in Sports Performance, Recreation, Innovation and Technology (SPRINT), 4960-320 Melgaço, Portugal; 8 Instituto de Telecomunicações, Delegação da Covilhã, 1049-001 Lisbon, Portugal; 9 The Research Centre in Sports Sciences, Health Sciences and Human Development (CIDESD), 5001-801 Vila Real, Portugal; 10 Department of Exercise Physiology, Faculty of Educational Sciences and Psychology, University of Mohaghegh Ardabili, 5619911367 Ardabil, Iran; 11 Faculty of Sport Sciences, University of Extremadura, 10003 Cáceres, Spain; 12 Department of Motor Performance, Faculty of Physical Education and Mountain Sports, Transilvania University of Braşov, 500068 Braşov, Romania

**Keywords:** Football, Metabolic power output, Positional differences, Match halves

## Abstract

**Objective:**

The aim of this study was (i) provide reference data of metabolic power-based measures during professional football matches; and to (ii) analyze the between-position and between-halves differences of power-based measures during professional football matches.

**Methods:**

Forty-six professional male players from two Turkish Super League teams were observed during two seasons, and 58 matches were analyzed. Total distance, equivalent distance, Low Power (LP), Intermediate Power (IP), High Power (HP), Elevated Power (EP), Max Power (MP) and power metabolic measures P_met_ at different match moments were considered.

**Results:**

Significant between-position differences were observed for IP (p: 0.000; d: 0.284), HP (p ≤ 0.001; d = 0.45), EP (p ≤ 0.001; d = 0.44), and for MP (p ≤ 0.001; d = 0.56), with the central defenders (CD) showing the lower values, and the central midfielders (CM) showing the higher values for the overall measures.

**Conclusion:**

Power-based measures are dependent on playing positions. While the CD have lower P_met_ values when compared to all positions, the CM have the greatest values. Training and recovery strategies must be ensured for CM players, especially those who have greater match participation.

## Background

Professional football match demands can be quantified through the use of multiple camera match analysis systems [1]. These practice have led to extensive knowledge regarding the players’ movements on the football pitch [2]. The majority of the studies that analyzed the running demands of football matches and training of elite football players has focused mainly on the distances covered at different speed thresholds [3]. However, more recently, researchers also expanded the knowledge regarding the acceleration and deceleration profiles of elite football players, that are considered relevant measures for the most crucial moments of a football match [4]. The use of both multiple cameras, GPS systems and other inertial measurement units allows coaches to analyze different speed thresholds, accelerations, decelerations and estimated metabolic power output (P_met_) measures [5–7].

Notwithstanding the available extensive data regarding time-motion analysis based on distances covered at various speed thresholds and accelerations/decelerations during football matches, they can potentially underestimate the P_met_ demands of running speeds thresholds that were previously labeled as “low- and high-intensity” speeds [8]. That is, a labeled “low-intensity” running speed can produce similar metabolic power demands of a “high-intensity” speed. The P_met_ measure is obtained through the multiplication of an estimated energy cost of both accelerations and decelerations performed on a horizontal level by the instantaneous running speed [6, 8]. The P_met_ calculation is based on the premise that the acceleration phase of running on a flat field is equivalent to run on uphill at a constant speed, in terms of energetic cost, which allows to estimate the P_met_ measure [9]. The use of speed, acceleration/deceleration and P_met_ measures extracted from GPS devices, can potentially be more relevant than the use of different speed thresholds in isolation, as intermittent changes in speed and direction, typical from team sports, increase its related energy cost [1].

Extensive research exists regarding the investigation of displacement measures of football players during official matches, which made possible to make further between-position comparisons [10–12]. It was previously shown that midfielders and wide attackers cover greater accelerations/decelerations and high-metabolic distances than the rest of outfield positions [13–15]. However, the majority of the available research focused on different speed thresholds and acceleration/deceleration measures to assess football match demands, with less focus on P_met_ measures [6, 15]. Indeed, there is a lack of studies focusing on different P_met_ thresholds such as the low power (LP: 0 to 10 W/kg), intermediate power (IP: 10 to 20 W/kg), high power (HP: 20 to 35 W/kg), elevated power (EP: 35 to 55 W/kg) and maximum power (MP), which may give better insights regarding match intensity [16]. Hoppe and colleagues [16] analyzed the differences in the above-mentioned P_met_ measures among playing positions of 12 professional football players, and found that there were no significant differences between playing positions for any P_met_ measures. As the authors of the above-mentioned study [16] stated, their findings contrast previous research that showed the existence of significant between-position differences in different speed thresholds [17, 18].

The quantification of such P_met_ measures through the use of GPS systems for monitoring the locomotor intensity of football matches has been questioned regarding its usefulness [19]. In fact, it was demonstrated that GPS-derived P_met_ measures largely underestimated the energy demands of football drills, and revealed poor reliability for P_met_ measures above 20 W/kg [19]. However, in response to the above-mentioned study [19], Osgnach et al. [20] stated that GPS-derived P_met_ measures can be used if the sampling frequency is above 10 Hz, otherwise, any other GPS system below 10 Hz is not recommended. Moreover, a recent study conducted on 17 professional football players compared the indirect estimation of a 10 Hz sampling frequency GPS-derived P_met_ through two different formulas with a direct measurement of P_met_ (P_VO2_) [7]. Interestingly, the mentioned study revealed that both GPS-derived P_met_ formulas and direct measurements were similar, demonstrating that this is a representative metabolic formula that is optimized to professional football contexts [7].

To the best of the authors’ knowledge, only one study examined the between-position differences of P_met_-based measures in professional football players [16]. However, the mentioned study analyzed only 12 players during five pre-season matches, which may not be a sufficient sample to generalize their findings. Also, no study examined the between-halves differences of P_met_-based measures. Moreover, given the above-mentioned relevance of using P_met_-based measures in professional football context, and that the use of estimated P_met_ through video match analysis using the same formulas and algorithms extracted from GPS systems may represent one step forward to the evolution of football demands monitoring [7], the present study aimed to (i) provide reference data of metabolic power-based measures during professional football matches; and to (ii) analyze the between-position and between-halves differences of power-based measures during professional football matches.

## Methods

### Study design and experimental approach

In this study, the data of 46 top elite football players were examined. All of the athletes were in the Turkish Super League. Athletes trained 6 days a week in a professional club environment. They participated in competitions every weekend. The matches played every weekend were recorded with the Sentio Sports optical tracking system. This study was approved by the university of Yalova committee Ethics Committee (Approval Number: 2022–5). The entire work follows the Declaration of Helsinki for the Humanities.

### Data collection and measurement

The data obtained from in the athletes were collected through optical cameras. These optical camera systems were working with the Sentio Sports optical tracking system. Optical cameras had a resolution of 4 K. Optical cameras and Sentio sports software operated via laptop. It has been reported that the Sentio system provides valid and reliable data in previous study [21]. Using this system, the positions of the players on the real field can be calculated with a margin of error of approximately 30–65 cm [21].

After the cameras were connected to the computer, he checked the data obtained by adjusting the sharpness and calibration on the field image of the cameras via Sentio software. After device and software installation, data was obtained through a technician.

### Data

The dataset contains football players’ match performance metrics of the 2019–2020 and 2020–2021 seasons from two Turkish Super League teams gathered from 58 matches. Only outfield players who participated for 90 min in a match were considered in the dataset. After filtering out observations that don’t meet the required criteria, a total of 387 observations for 46 different players remained in the dataset. Players are divided into 5 positions namely central defenders (CD, n = 10), full-backs (FB, n = 8), central midfielders (CM, n = 15), wide midfielders (WM, n = 5) and forwards (FW, n = 8).

In this dataset, metabolic power-related variables were considered. Total distance (TD) (m), equivalent distance (ED) (W/kg), Low Power (LP) (from 0 to 10 W/kg), Intermediate Power (IP) (from 10 to 20 W/kg), High Power (HP) (from 20 to 35 W/kg), Elevated Power (EP) (from 35 to 55 W/kg), Max Power (MP) (> 55 W/kg) [8], P_met_ (W/kg) in minutes (0–15; 15–30; 30–45; 45 + ; 60–75; 75–90; 90 + min), P_met_ at the first half (W/kg) (P[1st Half]), P_met_ at the second half (W/kg) (P[2nd Half]), and P_met_ at the whole match (W/kg) (P[Match]) were considered for further analysis.

### Statistical analysis

Shapiro-Wilks normality test confirmed that all the variables are normally distributed. Differences in distance, power, and metabolic power-related variables among each position are conducted with a one-way ANOVA analysis. If any significant difference occurs, then the source of difference is determined with the Tukey Honestly Significant Difference (HSD) test. Descriptive statistics and Cohen’s η^2^ effect sizes are also reported. The effect sizes are considered as small (η^2^ ≥ 0.01), medium (η^2^ ≥ 0.06), and large (η^2^ ≥ 0.14) based on benchmarks suggested by Cohen [22]. p values less than 0.05 are considered significant. All the statistical analysis is conducted in the R programming language [23].

## Results

There are 46 football players included in the study whereas 10 of them are central defenders, 8 of them are full backs, 15 of them are central midfielders, 5 of them are wide midfielders and 8 of them are forwards. The mean and standard deviation of players are 30.17 ± 3.97 years for the age, 180.46 ± 6.83 cm for the height, 75.62 ± 7.71 kg for the weight and variable 23.11 ± 1.11 kg/m^2^ for the Body Mass Index (BMI). Position specific descriptive statistics for the anthropometric variables is given in Table 1.

**Table 1:** Sample characteristics and anthropometric info of players

	All Players (n = 46)				Central Defenders (n = 10)			
	Age	Height (cm)	Weight (kg)	BMI (kg/m^2^)	Age	Height (cm)	Weight (kg)	BMI (kg/m^2^)
Min	22	167	60	20.28	25	183	76	21.50
Mean	30.17	180.46	75.62	23.11	30.1	188.10	82.8	23.41
St. Deviation	3.97	6.83	7.71	1.22	3.96	3.75	4.87	1.48
Median	31	181	75.5	23.18	30.5	188	84	23.39
Max	37	196	90	26.01	35	196	90	26.01

	Full Backs (n = 8)				Central Midfielders (n = 15)			
	Age	Height (cm)	Weight (kg)	BMI (kg/m^2^)	Age	Height (cm)	Weight (kg)	BMI (kg/m^2^)
Min	25	170	63	20.98	22	170	67	21.78
Mean	30.13	176.63	70.83	22.66	29.87	177.4	72.69	23.19
St. Deviation	3.36	3.99	5.91	1.086	4.79	5.11	4.29	0.83
Median	31	177	72	22.99	30	178	72	23.18
Max	35	182	78	23.97	37	187	82	25.26

	Wide Midfielders (n = 5)				Forwards (n = 8)			
	Age	Height (cm)	Weight (kg)	BMI (kg/m^2^)	Age	Height (cm)	Weight (kg)	BMI (kg/m^2^)
Min	27	167	61	21.29	25	172	60	20.28
Mean	29.2	174.4	68.6	22.52	31.5	184.25	79.38	23.31
St. Deviation	2.39	5.50	6.35	1.21	4.24	5.75	8.81	1.58
Median	28	175	70	22.34	32.5	185	80.5	23.57
Max	33	182	75	24.49	36	190	90	25.19

BMI: Body Mass Index

The mean and standard deviation for all the variables among each position is given in Table 2. Table 2 also contains ANOVA test results and Cohen’s η^2^ effect sizes results. The source of difference column summarizes pairwise comparison results from the Tukey HSD test.

**Table 2:** Descriptive statistics, and ANOVA results for power across positions

Variables	CD (n = 103)	CM (n = 84)	FB (n = 104)	FW (n = 48)	WM (n = 48)	F	p	Source of Difference	Effect Size
TD (m)	9574.01 ± 503.91	11,095.19 ± 702.76	10,342.76 ± 753.70	10,484.56 ± 737.11	10,503.84 ± 643.59	62	0.00	CD–FB, CM, WM, FW; CM–FB, WM, FW	0.394 (Large)
ED (W/kg)	11,084.54 ± 603.03	12,956.25 ± 900.49	12,162.3 ± 970.31	12,406.34 ± 849.32	12,421.21 ± 762.95	64.59	0.00	CD–FB, CM, WM, FW; CM–FB, WM, FW	0.403 (Large)
LP (0 to 10 W/kg)	4797.64 ± 317.24	4689.05 ± 295.76	4700.64 ± 298.16	4860.96 ± 304.19	4898.24 ± 279.44	6.371	0.00	FB, CM–WM, FW	0.063 (Moderate)
IP (10 to 20 W/kg)	2723.74 ± 322.29	3322.84 ± 325.1	2980.89 ± 368.18	2911.64 ± 349.12	2903.42 ± 293.08	37.88	0.00	CD–FB, CM, WM, FW; CM–FB, WM, FW	0.284 (Large)
HP (20 to 35 W/kg)	1343.51 ± 180.85	2009.81 ± 346.47	1610.77 ± 266.63	1580.58 ± 266.13	1575.64 ± 201.93	76.84	0.00	CD–FB, CM, WM, FW; CM–FB, WM, FW	0.446 (Large)
EP (35 to 55 W/kg)	483.29 ± 82.67	772.97 ± 151.31	690.47 ± 130.66	709.47 ± 123.76	699.06 ± 117.42	76.27	0.00	CD–FB, CM, WM, FW; CM–FB, WM, FW	0.444 (Large)
MP (W/kg)	225.17 ± 49.45	300.01 ± 67.44	358.58 ± 77.86	420.61 ± 65.25	426.31 ± 73.89	118.9	0.00	CD–FB–CM; WM, FW–CD, FB, CM	0.555 (Large)
P_met_ (W/kg) [0–15 min]	9.56 ± 0.77	11.28 ± 0.89	10.34 ± 0.91	10.82 ± 0.96	10.64 ± 0.9	48.94	0.00	CD–FB, CM, WM, FW; CM–FB, WM, FW; FB–FW	0.339 (Large)
P_met_ (W/kg) [15–30 min]	8.7 ± 0.82	10.09 ± 1.09	9.43 ± 1.18	9.83 ± 0.99	9.69 ± 0.85	24.8	0.00	CD–FB, CM, WM, FW; FB–CM	0.206 (Large)
P_met_ (W/kg) [30–45 min]	8.82 ± 0.84	10.42 ± 0.93	9.61 ± 1.13	9.61 ± 1.1	9.92 ± 0.87	32.23	0.00	CD–FB, CM, WM, FW; CM–FB, WM, FW	0.252 (Large)
P_met_ (W/kg) [45 + min]	8.39 ± 2.07	10.02 ± 2.58	9.52 ± 2.66	9.28 ± 2.67	9.77 ± 2.85	5.673	0.00	CD–FB, CM, WM	0.056 (Small)
P_met_ (W/kg) [1st Half]	9 ± 0.51	10.57 ± 0.71	9.79 ± 0.8	10.05 ± 0.71	10.06 ± 0.66	65.33	0.00	CD–FB, CM, WM, FW; CM–FB, WM, FW	0.406 (Large)
P_met_ (W/kg) [45–60 min]	8.87 ± 0.73	10.41 ± 1.3	9.81 ± 1.22	10.08 ± 1.23	9.99 ± 1.13	24.82	0.00	CD–FB, CM, WM, FW; FB–CM	0.206 (Large)
P_met_ (W/kg) [60–75 min]	8.33 ± 0.89	9.86 ± 1.08	9.31 ± 1.1	9.53 ± 1.12	9.49 ± 1.05	28.82	0.00	CD–FB, CM, WM, FW; FB–CM	0.232 (Large)
P_met_ (W/kg) [75–90 min]	8.19 ± 0.87	9.65 ± 0.89	9.12 ± 1.13	9.24 ± 0.92	9.41 ± 0.85	31.8	0.00	CD–FB, CM, WM, FW; FB–CM	0.25 (Large)
P_met_ (W/kg) [90 + min]	8.87 ± 1.68	9.75 ± 1.79	9.78 ± 1.89	9.48 ± 1.69	9.78 ± 1.69	4.613	0.00	CD–FB, CM, WM	0.046 (Small)
P_met_ (W/kg) [2nd Half]	8.49 ± 0.52	9.95 ± 0.75	9.43 ± 0.8	9.6 ± 0.78	9.64 ± 0.63	58.22	0.00	CD–FB, CM, WM, FW; CM–FB, FW	0.379 (Large)
P_met_ (W/kg) [Match]	8.74 ± 0.42	10.25 ± 0.67	9.6 ± 0.7	9.82 ± 0.67	9.84 ± 0.53	79.53	0.00	CD–FB, CM, WM, FW; CM–FB, WM, FW	0.454 (Large)

TD: total distance; ED: equivalent distance; LP: low power; IP: intermediate power; HP: high power; EP: elevated power; MP: max. power; P_met_: metabolic power

According to ANOVA results, all the variables showed differences for at least one position (p < 0.001). Mostly large effect sizes (η^2^ ≥ 0.14) are detected except for the P [45+] and P [90+] where small effect sizes (η^2^ = 0.056 and η^2^ = 0.46) are noted and except for LP where moderate effect sizes are found (η^2^ = 0.063).

The source of the difference column states the pairwise differed positions according to the Tukey HSD test. For the total distance, equivalent distance, intermediate power, high power, and elevated power, center backs have significantly lower averages compared to all other positions (for all pairwise comparisons p > 0.0001) and center midfielders have significantly higher averages compared to full-backs, wide midfielders, and forwards (for all pairwise comparisons p > 0.0001). Both full-backs and central midfielders have significantly lower averages compared to wide midfielders and forwards (for all pairwise comparisons p > 0.0001). For max power, central defenders, full-backs, and central midfielders are differentiated from each other (for all pairwise comparisons p > 0.0001) where central defenders have the lowest max power and full-backs have the highest max power. The remaining two positions, wide midfielders and forwards have significantly higher max power averages.

Metabolic power in the first fifteen minutes of the game significantly differs among positions. Central defenders have significantly lower values and central midfielders have significantly higher values compared to all other positions (for all pairwise comparisons p > 0.0001). Also, full-backs have significantly lower values than forwards (p < 0.0001).

Metabolic power in the minutes between 15 and 30 revealed that central defenders have significantly lower values compared to other positions (CM: p < 0.0001, FB: p < 0.0001, FW: p < 0.0001, WM: p < 0.0001). Also, full-backs have significantly lower average values compared to central midfielders (p = 0.001).

Metabolic power values for the last 15 min of the first half revealed central defenders and central midfielders are differentiated from other positions (for all pairwise comparisons p > 0.0001) where central defenders have the lowest averages and central midfielders have the highest averages.

The analysis for the additional time in the first half revealed central defenders have significantly lower metabolic power than full-backs (p = 0.012), central midfielders (p = 0.001), and wide midfielders (p = 0.016).

Looking at the first half altogether, central midfielders have significantly higher (CD: p < 0.0001, FB: p < 0.0001, FW: p = 0.0002, WM: p = 0.0003) and central defenders have significantly lower metabolic power compared to other positions (CM: p < 0.0001, FB: p < 0.0001, FW: p < 0.0001, WM: p < 0.0001).

The metabolic power differences between the minutes [45–60], [60–75], and [75–90] detect the same results. For all three time intervals, central defenders have significantly lower values compared to all other positions (for all pairwise comparisons p > 0.0001). Also, full-backs have significantly lower metabolic power compared to central midfielders (for all pairwise comparisons p > 0.0001).

For the additional time of the second half central defenders showed a difference compared to full-backs (p = 0.002), central midfielders (p = 0.007), and wide midfielders (p = 0.028).

For the whole second half, central defenders have lower averages compared to all other positions (CM: p < 0.0001, FB: p < 0.0001, FW: p < 0.0001, WM: p < 0.0001) and central midfielders have higher averages compared to full-backs (p < 0.0001) and forwards (p = 0.044).

Central defenders have lower metabolic power (CM: p < 0.0001, FB: p < 0.0001, FW: p < 0.0001, WM: p < 0.0001) and central midfielders have higher metabolic power (FB: p < 0.0001, FW: p = 0.0008, WM: p = 0.002) compared to all other positions for the whole match.

## Discussion

The present study aimed to determine the classified metabolic power measures of professional football players according to playing positions and periods of match duration. The major findings were that (1) the central midfielders generally covered the highest distances at all P_met_-based distance measures [ED (W/kg), IP (10–20 W/kg), HP (20–35 W/kg), EP (35–55 W/kg)] except for LP (0–10 W/kg) and MP (> 55 W/kg). The central defenders covered the lowest P_met_-based distances except for LP (0–10 W/kg). (2) While the players covered mostly P_met_-based distances in the first half of the match, they covered the highest distances in the first 0–15 min. period and the lowest distances in the 75–90 min. period.

To date, when examining the activity profile and external loads of a football match, the distance covered in different speed zones or the time spent in these zones were generally taken into account. However, current researches show that ignoring the energy demands associated with acceleration and deceleration causes an underestimation of the total energy cost [24, 25]. But metabolic power estimates can better inform coaches and sports scientists about energy cost and can be useful for improving specific fitness in football [26]. In the present study, when comparing all P_met_-based distance measures according to playing positions, distances were higher for central midfielders at IP (10–20 W/kg), HP (20–35 W/kg), and EP (35–55 W/kg) zones. Also, wide midfielders covered the highest distances at LP (0–10 W/kg) and MP (> 55 W/kg) zones (Table 1). The lowest distances for the central defenders were observed in all zones except for the LP (0–10 W/kg) zone. The results of our research are supported by metabolic power-based researches on game positions. However, the sample group and the number of the examined matches, and the quality of the matches make our study more comprehensive. Manzi et al. reported that while midfielders covered the highest distances, central backs covered the lowest distances. But in this previous study, only one P_met_-based running zone (> 25 W kg^−1^) was measured, and some positions (ex: midfielders) not been detailed [27].

Gaudino et al. reported that P_met_ measures (P_met_ = Mean metabolic power; HP = High P_met_ (20–35 W kg^−1^); EP = Elevated P_met_ (35–55 W kg^−1^) were greater in central midfielders compared to all other positions. Contrary to our results, Hoppe et al. stated that there were no differences between player positions in their P_met_-based measurements [16]. However, in this study, the small sample size (n = 12), the fact that the players were classified in only three positions (defenders, midfielders, and attackers), and the measurements were collected in only five pre-season games draw attention as quite limiting factors. Studies have shown that the running activity profile differs in detailed playing positions [28, 29] and there are differences in metabolic power metrics between friendly matches and official matches [30]. The game position differences in the results of our study can be associated with the tactical roles of the players. For example, central midfielders perform more power events due to their central role between the offensive and defensive areas on the football pitch. Similarly, as the results of our study showed (Maximal P_met_ (> 55 W kg^−1^) wing midfielders also have to display higher power events due to their attacking roles.

Another finding of this study was that metabolic P_met_-based running distances differed according to the periods of the football match. To the best of the authors' knowledge, this is the first study to examine metabolic P_met_-based runs in periods throughout the match. It was observed that the players covered higher P_met_-based distances in the second half of the matches compared to the first half. While the highest running distances were measured in the 0–15 min period, the lowest P_met_-based distances were measured in the 75–90 min period. In addition, the central midfielders reached the highest values in all halves and periods compared to other positions, while the central defenders had the lowest values (Table 1). The metabolic power approach takes into account the athlete's acceleration for a more complete assessment of the demands of the field sport by including the energy cost [30]. However, considering the 15-min periods, high-intensity movements and the indicators of acceleration and deceleration capacity decrease towards the end of the football match period [31]. The results of a previous study examining acceleration and deceleration-based runs classified as intensity in 15-min periods of a football match also show that a decrease in central nervous drive and an increase in peripheral fatigue may cause a decrease in running distances towards the end of the match [32]. Again, tactical approaches and the state of playing the match may require players from different positions to be more active. In an offensive-heavy match against a weak opponent, central defenders can perform less intense activities and runs. Similarly, offensive players may find fewer positions when playing against a strong team. However, central midfielders have to be active throughout the match in any case. For this reason, the decrease in high-intensity activities towards the end of the match due to fatigue and the effect of tactical situations on the activity of playing positions may cause differences between periods. The small differences in the + 90 min period maybe because this period is different for each match.

## Conclusion

In this study, power-based measures were dependent on different field positions. The CD had the lowest values for the overall power-based measures, while the CM had the greatest values. Interestingly, the analysis of the different periods of the 1st and 2nd match halves showed that both CD and CM remained with the lowest and the greatest values for TD and the overall power-based measures, respectively. For these reasons, coaches should consider the effects of power measures in CM players, to ensure that athlete are being well prepared during training in order to withstand the P_met_ match demands.

## Data Availability

The data presented in this study are available on website: https://osf.io/rwex9/ with Identifier: 10.17605/OSF.IO/RWEX9
